# Innovatively Therapeutic Strategy on Lung Cancer by Daily Drinking Antioxidative Plasmon-Induced Activated Water

**DOI:** 10.1038/s41598-018-24752-x

**Published:** 2018-04-20

**Authors:** Chien-Kai Wang, Hsiao-Chien Chen, Sheng-Uei Fang, Chia-Wen Ho, Cheng-Jeng Tai, Chih-Ping Yang, Yu-Chuan Liu

**Affiliations:** 1 Department of Animal Science, National Chung Hsing University, No. 250, Guoguang Rd., Taichung, 402 Taiwan; 2 Division of Hematology and Oncology, Department of Internal Medicine, Taipei Medical University Hospital, No. 252, Wuxing St., Taipei, 11031 Taiwan; 3 Department of Biochemistry and Molecular Cell Biology, School of Medicine, College of Medicine, Taipei Medical University, No. 250, Wuxing St., Taipei, 11031 Taiwan; 4 Division of Gastroenterology and Hepatology, Department of Internal Medicine, Taipei Medical University Hospital, No. 252, Wuxing St., Taipei, 11031 Taiwan; 5 Department of Internal Medicine, School of Medicine, College of Medicine, Taipei Medical University, No. 250, Wuxing St., Taipei, 11031 Taiwan; 6 Center for Cancer Research, Taipei Medical University, No. 250, Wuxing St., Taipei, 11031 Taiwan

**Keywords:** Cancer therapy, Nanomedicine

## Abstract

Many human diseases are inflammation-related, such as cancer and those associated with aging. Previous studies demonstrated that plasmon-induced activated (PIA) water with electron-doping character, created from hot electron transfer via decay of excited Au nanoparticles (NPs) under resonant illumination, owns reduced hydrogen-bonded networks and physchemically antioxidative properties. In this study, it is demonstrated PIA water dramatically induced a major antioxidative *Nrf2* gene in human gingival fibroblasts which further confirms its cellular antioxidative and anti-inflammatory properties. Furthermore, mice implanted with mouse Lewis lung carcinoma (LLC-1) cells drinking PIA water alone or together with cisplatin treatment showed improved survival time compared to mice which consumed only deionized (DI) water. With the combination of PIA water and cisplatin administration, the survival time of LLC-1-implanted mice markedly increased to 8.01 ± 0.77 days compared to 6.38 ± 0.61 days of mice given cisplatin and normal drinking DI water. This survival time of 8.01 ± 0.77 days compared to 4.62 ± 0.71 days of mice just given normal drinking water is statistically significant (*p* = 0.009). Also, the gross observations and eosin staining results suggested that LLC-1-implanted mice drinking PIA water tended to exhibit less metastasis than mice given only DI water.

## Introduction

Cell inflammation is an early expression in the progression of many chronic diseases including Alzheimer’s disease^1,2^, chronic kidney disease^3,4^, and various cancers^5,6^, as well as conditions related to aging^7,8^. As shown in the literature^9,10^, reactive oxygen species (ROS) are strongly associated with chronic inflammation and cancer. Oxidative stress is predominantly caused by the accumulation of ROS and is distinguished by inflamed tissues. Ohsawa and colleagues reported a method utilizing dissolved hydrogen to selectively depress hydroxyl radicals in cells to reduce damage to cells by ROS^11^. On the other hand, hot-electron-mediated surface chemistry with efficient energy transfer based on noble metal nanoparticles (NPs) with well-defined localized surface plasmon resonance (LSPR) bands is garnering wide attention. The created chemicurrent at excited metal NPs can catalyze surface reactions of CO oxidation or hydrogen oxidation^12,13^. In addition, photothermal ablation based on Au nanorods was employed to effectively kill cancer cells^14^. In our previous report^15^, hot electron transfer (HET) on supported AuNPs was innovatively utilized to create plasmon-induced activated (PIA) water with reduced intermolecular hydrogen bonds (HBs). The created liquid water in a hot-electron-doping state possesses a unique ability to scavenge free hydroxyl and 2,2-diphenyl-1-picrylhydrazyl (DPPH) radicals and to effectively reduce nitric oxide (NO) release from lipopolysaccharide (LPS)-induced inflammatory cells. These distinct properties show promise for its innovative availability to increase the efficiency and safety of hemodialysis^16^.

The biological effects of PIA water currently remain unclear. The previous study indicated that PIA water produced by AuNPs can reduce NO release by LPS-treated monocytes^15^. This finding suggested that PIA water has*in vitro* antioxidative activity to prevent oxidative stress induced by acute inflammation. ROS are not only major contributors to oxidative stress but also play important roles in the progression of many diseases, including inflammation and cancers.^17^ PIA water also showed that cells defend against ROS-induced cell damage using various defense systems^18^. One of the most important mechanisms is the Kelch-like ECH-associated protein 1 (Keap1)/nuclear factor erythroid 2 related factor 2 (*Nrf2*)/antioxidant response element (ARE) pathway. The core factor of this pathway, *Nrf2*, is a redox-sensitive transcription factor which provides protective effects against oxidative stress. To evaluate the activation of the Keap1/*Nrf2*/ARE pathway by PIA water treatment*in vitro* may be helpful to further understand the antioxidative and anti-inflammation effects of PIA water.

Since PIA water exhibited anti-inflammatory activity *in vitro*, a preclinical mouse disease model is worthy of further study to evaluate the anti-inflammatory potential of PIA water in the chronic inflammation-related disease of non-small cell lung cancer (NSCLC). As shown in the literature, chronic inflammation and associated oxidative stress contribute to the carcinogenesis of NCSLC^19^. Administration of PIA water to NSCLC-bearing animals may mediate the inflammatory status of the tumor microenvironment and delay the progression of lung carcinoma cells. Therefore, these effects may benefit integration with conventional cancer chemotherapy to improve the tumor suppression efficiency of chemotherapeutic drugs. To explore potential clinical applications of PIA water in NSCLC therapy, a transpleural orthotopic mouse model using Lewis lung cancer-1 (LLC-1) cells (a cell line originally isolated from C57BL/6 mice) was applied to examine the antitumor effects of PIA water on LLC-1-implanted mice. This mouse lung cancer model is suitable to observe lung metastasis from the pleura and evaluate the antitumor efficiency of potential cancer therapeutic strategies^20^. The use of B6 mice with LLC-1 implantation maintains the complete immune capability compared to commonly applied immunodeficient mice. Also, this is an appropriate model for evaluating the potential antitumor effects of PIA water in normal physiological conditions. The antitumor effect of PIA water was examined both *in vitro* in LLC-1 cells and *in vivo* in LLC-1-implanted mice alone or with a conventional chemotherapy agent, cisplatin, which is currently the primary drug for NSCLC chemotherapy. Taken together, the aims of this study were to evaluate the potential benefits of PIA in chronic inflammation-related diseases using a mouse model. This study may provide useful information to explore probable clinical applications of PIA water.

## Results and Discussion

### Antioxidative activity of PIA water

**Figure 1:**
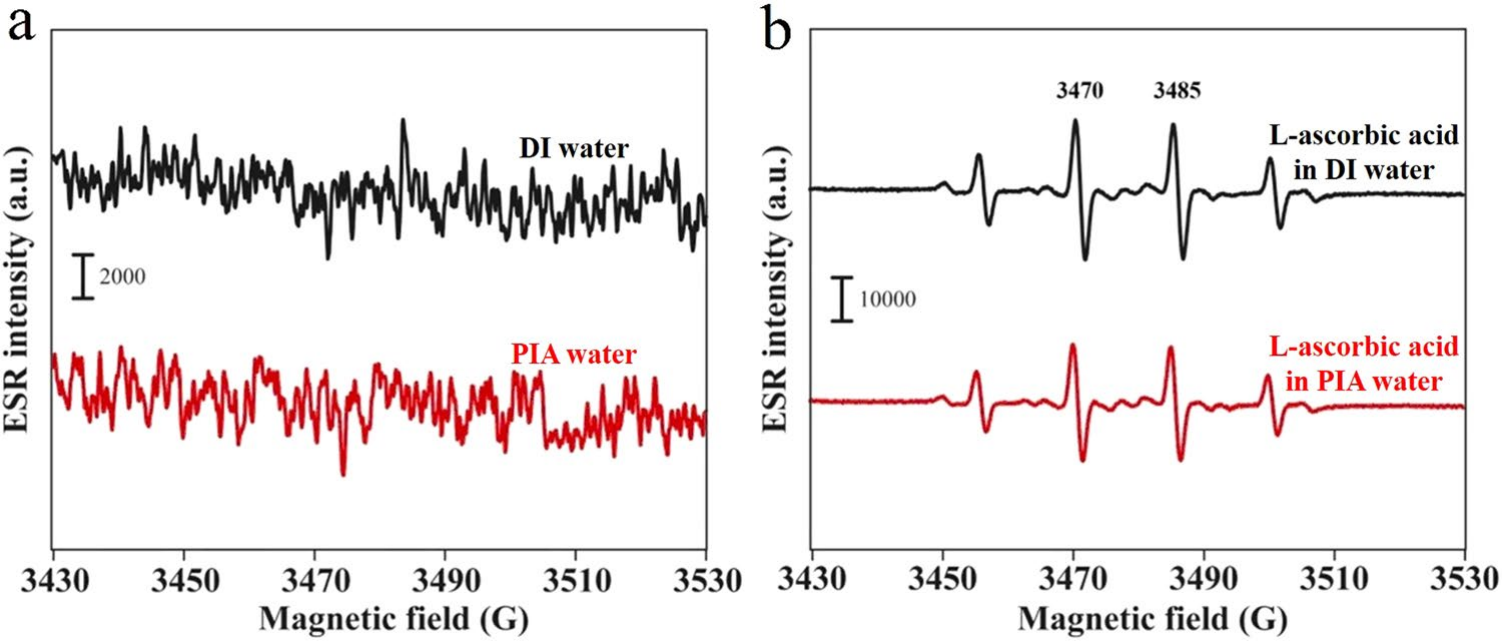
ESR spectra of hydroxyl free radicals based on DI water and PIA water. (**a**) Spectra of DI water (black line) and PIA water (red line) for reference. (**b**) Spectra of DI water plus the antioxidant L-ascorbic acid (black line) and PIA water plus L-ascorbic acid (1.775 µM) (red line). Hydroxyl free radicals were obtained using the well-known Fenton reaction, in which ferrous iron donates an electron to hydrogen peroxide to produce a hydroxyl free radical.

As reported in the literature, hydroxyl radicals are the most cytotoxic ROS and as such, they can directly or indirectly damage DNA and cause cancer^18,21,22^. It is well known that excessive amounts of ROS are produced at sites of inflammation. Therefore, the unique ability to scavenge free hydroxyl radicals and other distinct properties of PIA water compared to deionized (DI) water may offer a new therapy for suppressing inflammation and even for curing cancer. Figure 1a demonstrates the electron spin resonance (ESR) spectra regarding hydroxyl radicals of DI water and PIA water for reference. No significant peaks were observed for either DI or PIA water. This result suggests that the created electron-doping PIA water differs from the reported engineered water nanostructures with a very strong surface charge, which demonstrated strong signals of hydroxyl radicals in an ESR spectrum^23^. Figure 1b demonstrates the ESR spectra regarding hydroxyl radicals of DI water plus the known antioxidant, L-ascorbic acid^24^, and PIA water plus L-ascorbic acid, in the well-known Fenton reaction, as described in the experimental section. The four ESR splitting signals shown in these spectra are characteristic of hydroxyl radicals^11,24^. Interestingly, the production of hydroxyl radicals was significantly reduced in the PIA water-based system compared to the DI water-based system with L-ascorbic acid. The corresponding ESR average intensities of the two strongest peaks at ca. 3473 and 3488 G in the PIA water-based system significantly decreased by ca. 21% (**p < 0.01), compared to that for an experiment performed in the DI water-based system. Furthermore, in the Fenton reaction, free hydroxyl radicals are generated from hydrogen peroxide (H_2_O_2_). H_2_O_2_ is one of the products of reactions catalyzed by oxidase enzymes in many biological and environmental processes. However, H_2_O_2_ is also one kind of ROS that can cause functional and morphological disturbances as well as cancer when produced in excess in the human body. It was demonstrated H_2_O_2_ is as a reservoir for generating HOx by reacting with OH radicals (Eq. 1)^25,26^. Water was shown to be favorable for its catalytic effect on radical-radical (H_2_O_2_-OH) reactions due to the ability of water to form stable complexes (HO_2_•H_2_O) with HO_2_ radicals through hydrogen bonding.

1 $${{\rm{H}}}_{2}{{\rm{O}}}_{2}+{\rm{OH}}\to {{\rm{HO}}}_{2}+{{\rm{H}}}_{2}{\rm{O}}\,({\rm{in}}\,{\rm{the}}\,{\rm{atmosphere}})$$

2 $${{\rm{HO}}}_{2}+{{\rm{H}}}_{2}{\rm{O}}\leftrightarrow {{\rm{HO}}}_{2}\,\bullet \,{{\rm{H}}}_{2}{\rm{O}}\,({\rm{in}}\,{\rm{the}}\,{\rm{atmosphere}})$$

In the presence of liquid water, the oxidation of H_2_O_2_ becomes more complex by the following three steps^27^.

3 $${{\rm{H}}}_{2}{{\rm{O}}}_{2}\,\bullet \,{{\rm{H}}}_{2}{\rm{O}}+{\rm{OH}}\to {{\rm{HO}}}_{2}+2{{\rm{H}}}_{2}{\rm{O}}$$

4 $${{\rm{H}}}_{2}{{\rm{O}}}_{2}+{{\rm{H}}}_{2}{\rm{O}}\,\bullet \,{\rm{OH}}\to {{\rm{HO}}}_{2}+2{{\rm{H}}}_{2}{\rm{O}}$$

5 $${{\rm{H}}}_{2}{{\rm{O}}}_{2}\,\bullet \,{\rm{OH}}+{{\rm{H}}}_{2}{\rm{O}}\to {{\rm{HO}}}_{2}+2{{\rm{H}}}_{2}{\rm{O}}$$

**Figure 2:**
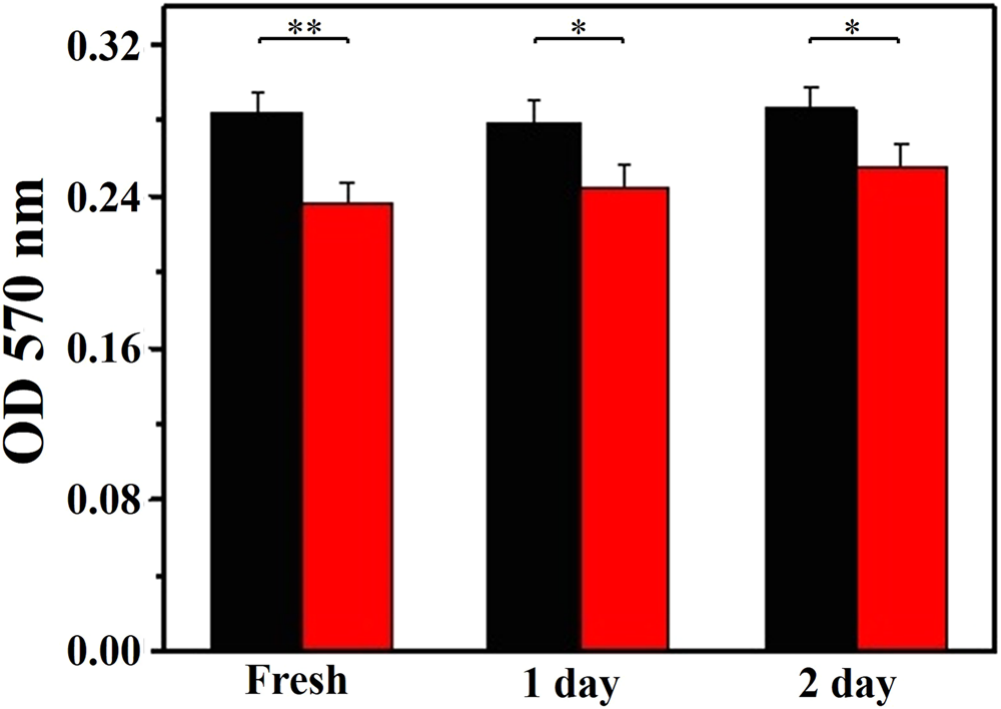
Antioxidative effect of PIA water to H_2_O_2_. The OD at 570 nm of H_2_O_2_ (2.5 nm) prepared in DI and PIA waters. The corresponding*p* values are 0.00491, 0.0233 and 0.0357 for PIA water after its preparation for 0, 1 and 2 days, respectively.

**Figure 3:**
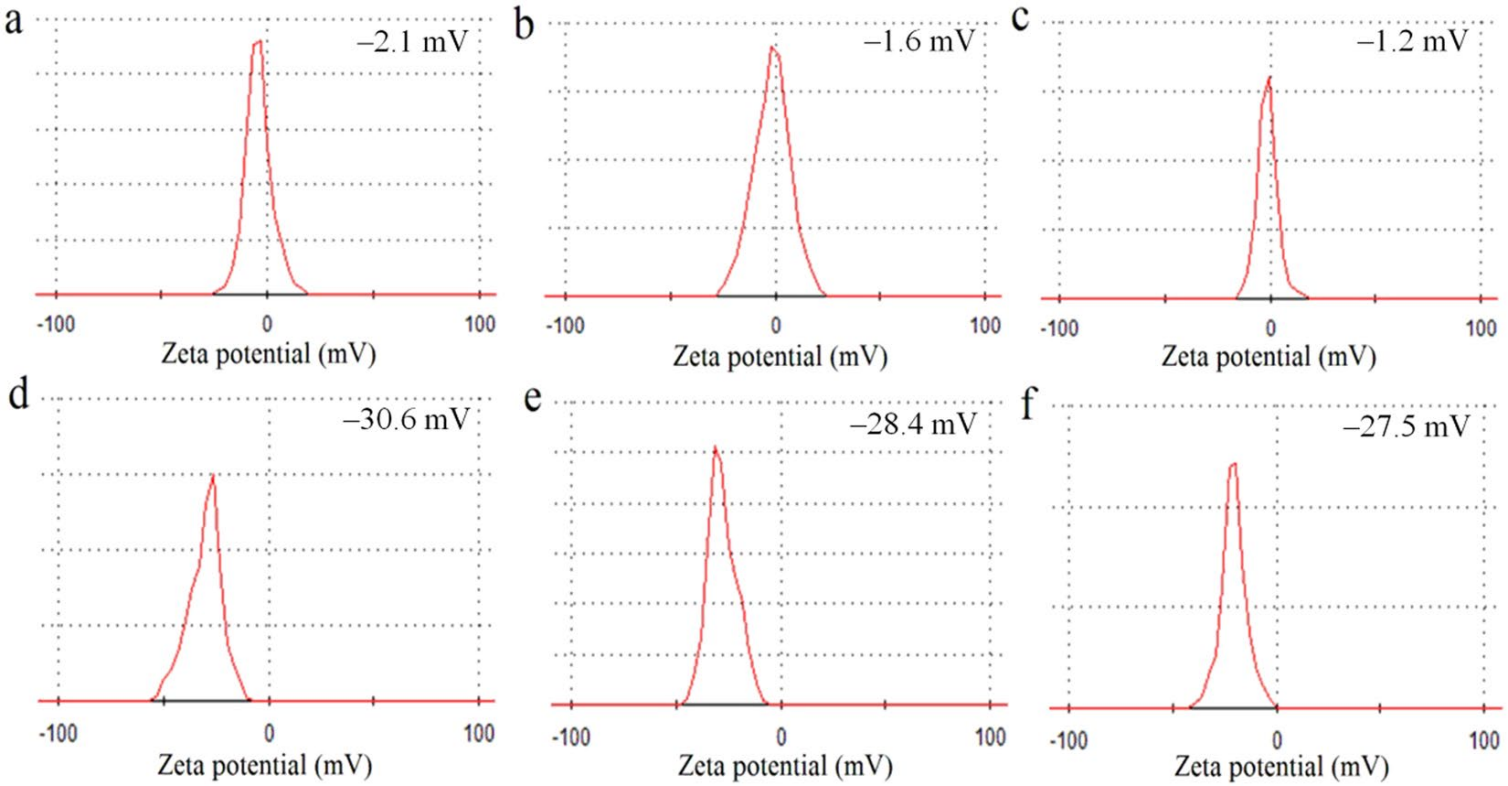
The stability of PIA water. The time-dependent zeta potentials of (**a**–**c**) DI and (**d**–**f**) PIA waters over time.

Either in the atmosphere or in an aqueous solution, water deeply dominates the equilibrium of these reactions. In a previous study, it was reported that PIA water provides more available sites for forming hydrogen bonds^15^. In addition, compared to bulk water which is recognized as being constructed of numerous large-sized water clusters, PIA water with reduced hydrogen bonds forms smaller water clusters, and thus presumably has more active sites. Therefore, according to Le Chatelier’s principle, the positive reactions of Eqs 2–5 dramatically occur accompanied by consumption of quantities of H_2_O_2_ and OH free radicals when DI water is replaced by PIA water. Based on the above reasons, PIA water might consume H_2_O_2_ during the Fenton reaction. The evidence of scavenging H_2_O_2_ by PIA water was examined using an H_2_O_2_ assay kit (Fig. 2). The optical density (OD) at 570 nm for H_2_O_2_ (2.5 nmol) prepared using DI water was 0.284 ± 0.010. This value decreased to 0.235 ± 0.011 as DI water was replaced by PIA water, meaning nearly 17.2% of the H_2_O_2_ had been consumed by PIA water. Also, the above ESR result demonstrated that PIA water plus L-ascorbic acid can reduce more than 21.0% of the hydroxyl radicals from the Fenton reaction than can DI water plus L-ascorbic acid. The source of hydroxyl radicals was from H_2_O_2_, and 17.2% of H_2_O_2_ was consumed by PIA water. In addition to the effect of PIA water on H_2_O_2_, PIA water plus L-ascorbic acid reduced more than 4.2% of the hydroxyl radicals than did DI water plus L-ascorbic acid. This means that a synergetic effect occurred between PIA water and L-ascorbic acid. To the best of our knowledge, this enhanced antioxidant activity of scavenging free radicals in PIA water-based system instead of a conventional DI water-based system is the first report in the literature. Additionally, the ability of PIA water to scavenge H_2_O_2_ weakened slightly with time. Also, it was found that the zeta potential of fresh PIA water was −30.6 mV, and it turned more positively to −28.4 and −27.5 mV after its preparation for 1 and 2 days, respectively, in storage. Meanwhile, the zeta potential of DI water did not clearly change. These time-dependent results indicated that PIA water was in a meta-stable state (Fig. 3). After one-day storage of PIA water in a capped container, the zeta potential was slightly changed from −30.6 mV to −28.4 mV (change by ca. 7.2%). In animal experiments, as-prepared drinking PIA water was also saved in a close container. It suggested that the activity of as-prepared PIA water was slightly decayed with time.

### Induction of antioxidative Nrf2 gene transcription by PIA water

**Figure 4:**
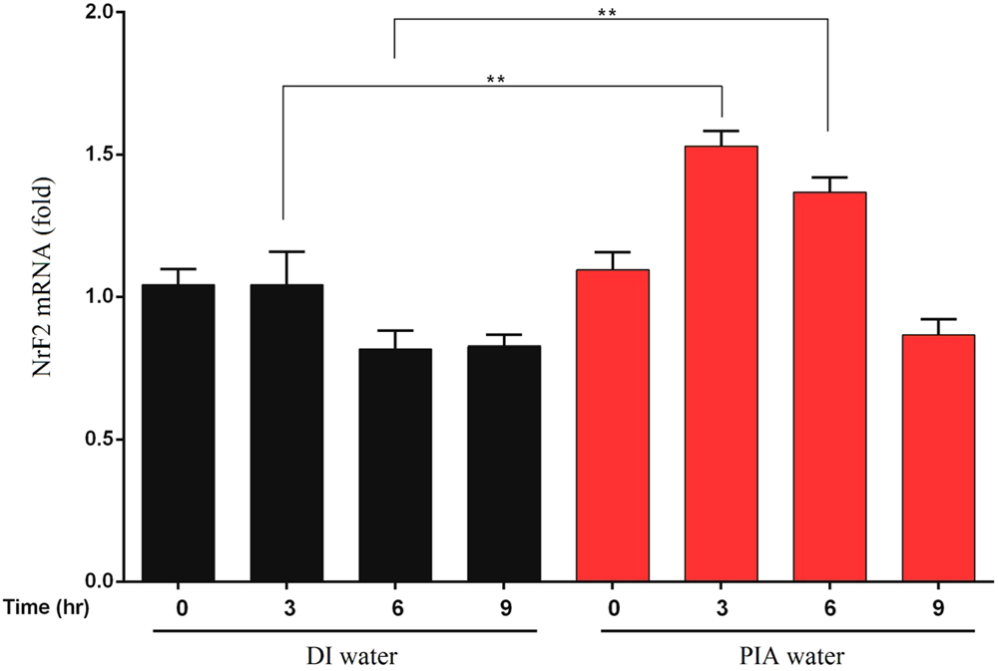
Induction of *Nrf2* expression in human gingival fibroblasts (HGFs) exposed to PIA water. HGFs were incubated in culture medium prepared with DI or PIA water for 0, 3, 6, and 9 h. *Nrf2* mRNA expression levels were quantified by a real-time PCR, and results are presented as the relative normalized expression with *GAPDH*. Data were analyzed by Student’s *t*-test, and results are presented as the mean ± SD. **p < 0.01. The corresponding *p* values are 0.00521 and 0.00453 for 3 and 6 hours, respectively.

Since *Nrf2* is an antioxidative gene that prevents damage from ROS, the role of PIA water on the *Nrf2* gene expression was investigated to examine the antioxidative property of PIA water. In experiments, human gingival fibroblasts (HGFs) were exposed to cultured media prepared by DI or PIA water for 0, 3, 6, and 9 h, then messenger (m)RNA expression levels of *Nrf2* were determined by a real-time polymerase chain reaction (PCR). As shown in Fig. 4, the mRNA expression levels of *Nrf2* in HGFs was significantly induced by PIA water with exposure for 3 to 6 h, and consequently decreased to a normal level after exposure for 9 h. This result suggests a potential role of PIA water on the oxidative stress defense through *Nrf2* gene induction.

A previous study showed that *Nrf2* is a transcription factor that responds to oxidative stress by binding to the ARE in the promoter of antioxidant enzyme genes such as NAD(P)H: quinone oxidoreductase 1, glutathione S-transferases, and glutamate cysteine ligase^10^. Activation of the *Nrf2* pathway by sulforaphane, a phytochemical, was well documented and linked to cancer chemoprevention^11^. Similarly, curcumin, a well-known polyphenol, was also reported to induce *Nrf2* and had an antioxidant response^12^. PIA water may have a similar property to these antioxidant substances. Therefore, the exact molecular mechanism based on PIA water requires further investigation.

Although inflammation is one of the major defense mechanisms against infection and in the repair of injured tissues, prolonged chronic inflammation may also contribute to the development of various chronic and neoplastic diseases in humans. The development of nanotechnology and nanomaterials with anti-inflammatory properties is rapidly being exploited, and the anti-inflammatory potential of PIA water is therefore worth of further evaluating. Therefore, we demonstrated that PIA water increased *Nrf2* expression, one of the defense mechanisms against the ROS-induced cellular stress response in HGFs. Additionally, administration of PIA water can be developed into an alternative strategy for treating chronic diseases such as NSCLC which is related to local chronic inflammation.

### PIA water treatment suppressed metastasis in LLC-1-grafted mice, and enhanced the overall survival in combination with cisplatin

**Figure 5:**
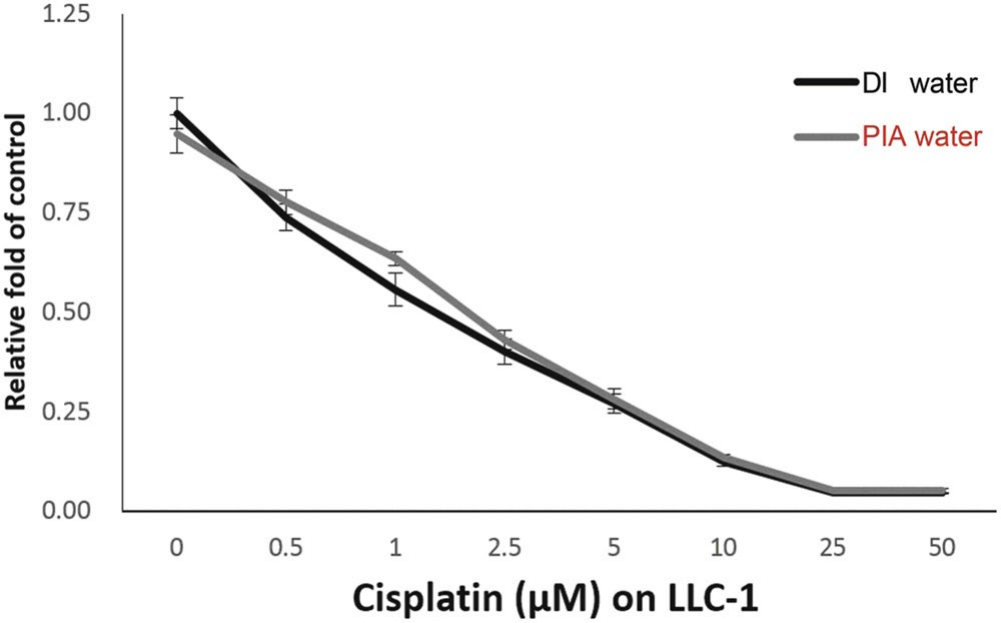
The *in vitro* experiment of LLC-1 cells treated with DI and PIA waters plus cisplatin. LLC-1 cells were treated with 0–50 µM cisplatin in DI or PIA water-prepared culture medium for 48 h. Cell viability was determined by an MTT assay, and data are presented as the mean ± SD.

**Figure 6:**
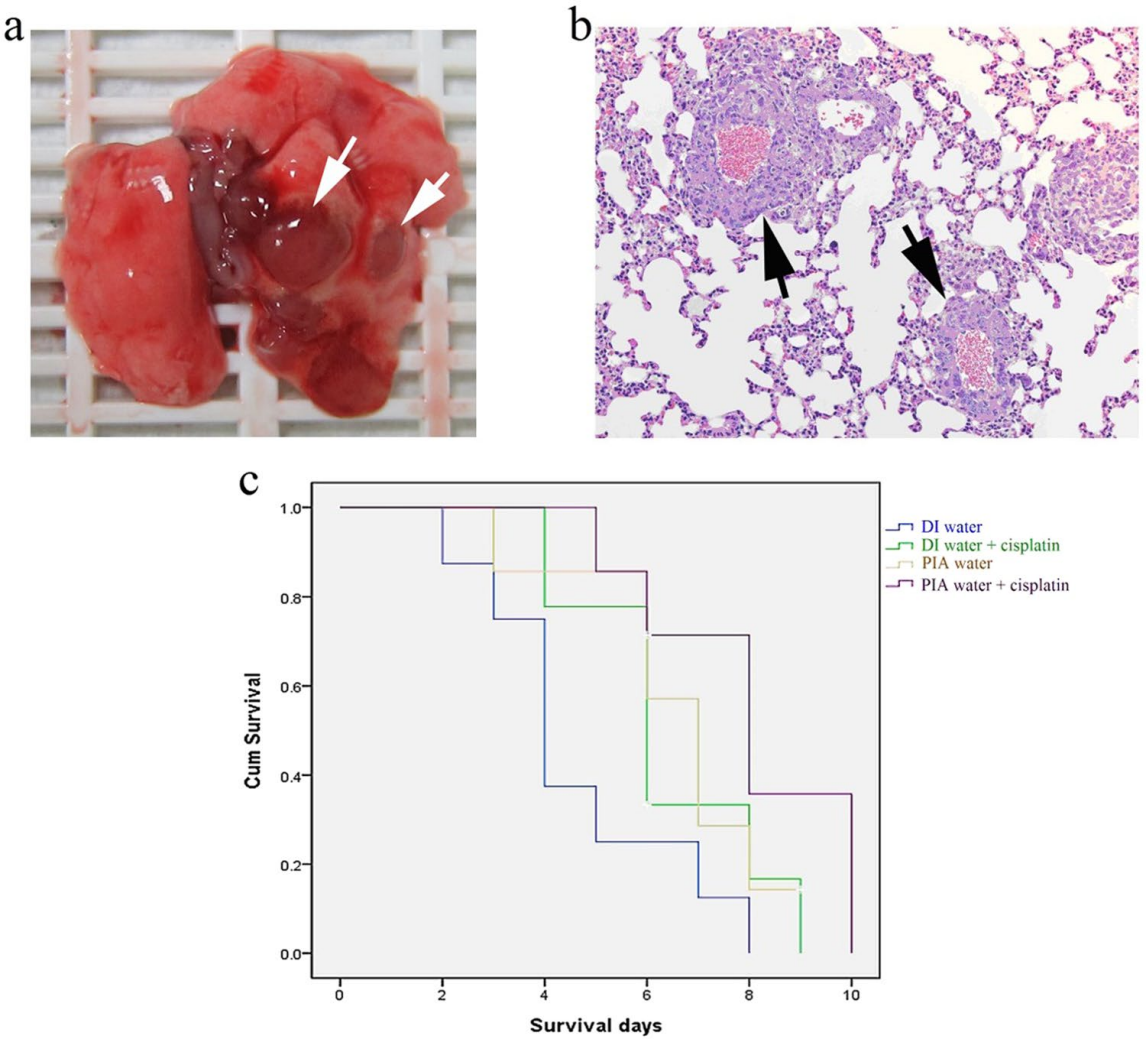
Pathological features and survival curve on the LLC-1-xenograft mice. (**a**) Lung metastasis in LLC-1-xenograft mice: gross observation of the whole lung (arrows). (**b**) Lung metastasis in LLC-1-implanted mice, HE staining (right, 200x magnification) of metastatic tumor lesions (arrows). (**c**) The overall survival time (days) of LLC-1-implanted mice treated with DI water (n = 9), DI water plus cisplatin (n = 8), PIA water (n = 7), or PIA water plus cisplatin (n = 7).

**Figure 7:**
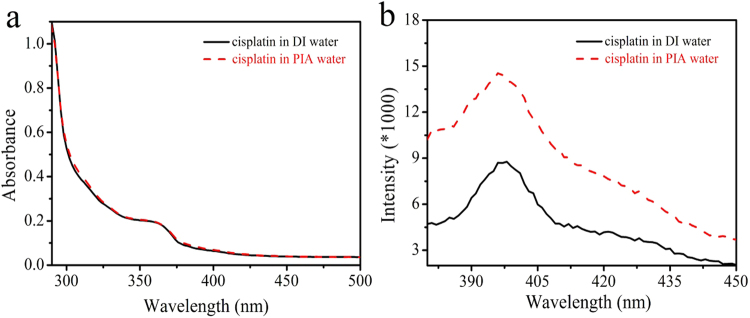
Conformation of cisplatin in DI and PIA waters. (**a**) The absorption spectra of cisplatin in DI and PIA waters. (**b**) The PL spectra of cisplatin in DI and PIA waters with an excitation wavelength of 350 nm.

**Figure 8:**
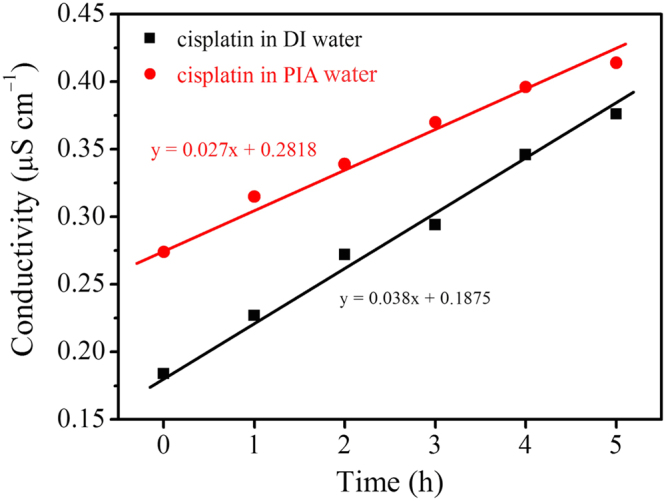
The conductivities of cisplatin solutions in DI and PIA water with time.

Before the preclinical test of PIA water in LLC-1-grafted mice, LLC-1 cells were incubated with DI water or PIA water with 0–50 µM cisplatin to examine whether PIA water affected the cell proliferation of LLC-1 alone or cytotoxicity of cisplatin toward LLC-1 cells *in vitro*. As shown in Fig. 5, PIA water incubation had no effect on LLC-1 cell proliferation compared to DI water, as neither influenced the cytotoxicity of cisplatin toward LLC-1 cells. These results suggested that PIA water may have no direct effect on LLC-1 cells *in vitro*. Furthermore, gross observations of whole lungs to lung metastasis in LLC-1 xenograft mice are shown in Fig. 6a. All tumor-like lesions were identified on lung lobes and thoracic walls but not presented in other organs of thoracic and abdominal cavities. These tumor-like lesions were further identified by hematoxylin and eosin staining as LLC-1 tumor lesions (Fig. 6b). As shown in Fig. 6b, the LLC-1 tumor lesions localized around blood vessels suggested that the injected LLC-1 cells invaded into pulmonary tissues *via* circulation. The metastasis rate of LLC-1 cells was calculated according to gross observations of the LLC-1 lung tumor presence and was analyzed by a two-tailed Fisher’s test. Interestingly, five of 17 LLC-1 grafted mice drinking DI water demonstrated lung metastasis compared to zero of 14 LLC-1 grafted mice drinking PIA water (Table 1). The metastasis rate in PIA water-consuming mice was significantly lower than that of DI water-consuming mice. The average survival time of PIA water-fed mice was 6.57 ± 0.66 days, whereas in DI water-fed mice, it was 4.62 ± 0.71 days. In cisplatin-administrated mice, PIA water-fed mice also had a prolonged survival time of 8.01 ± 0.77 days compared to 6.38 ± 0.61 days for DI water-fed mice. This result suggests that PIA water may enhance the tumor suppression efficiency of cisplatin in LLC-1-implanted mice. This can be attributed to the different state of cisplatin in DI and PIA waters. It was reported that cisplatin is poorly soluble in water^28^, indicating some aggregations of cisplatin molecules are generated in DI water. The absorption spectra showed the OD at 362 nm of cisplatin in PIA water was almost the same as that in DI water (Fig. 7a). However, a significant difference was observed in photoluminescence (PL) spectra with an excitation wavelength of 350 nm (Fig. 7b). Cisplatin displayed emission bands at 396 and 397 nm in DI and PIA waters, respectively. The PL intensity of cisplatin in PIA water was 1.6-fold higher than that in DI water. This evident difference perhaps can be attributed to the status of cisplatin complexes in the different waters. The poor solubility of cisplatin in DI water results in the formation of some aggregations that quenched the fluorescence. However, this phenomenon was not observed because cisplatin can be more easily dissolved in PIA water. The solubilities of cisplatin in DI and PIA water were measured at 25 °C. The solubility of cisplatin in PIA water was 3.4 ± 0.11 mg mL^−1^ which was higher than that in DI water (2.6 ± 0.01 mg mL^−1^). The increased solubility was ca. 30.8%, indicating PIA water improved the solubility of cisplatin. This reveals that PIA water improved the solubility of cisplatin and reduced interactions among cisplatin molecules, thus showing a higher PL intensity. Compared to the aggregated cisplatin in DI water which could be considered to be a large size and of high molecule weight, well-dispersed cisplatin in PIA water could be transported more easily across plasma membranes, thus enhancing the tumor suppressive efficiency of cisplatin in LLC-1-implanted mice. Furthermore, the zeta potentials of cisplatin solutions with 0.5% sodium chloride (NaCl) were also monitored over time (Fig. S1). Charges of the cisplatin solution were −8.6 and −19.3 mV with DI and PIA waters, respectively. Moreover, the negatively charged environment was stable for the following 2 days. A negatively charged environment is favorable for maintaining the activity of cisplatin before it is transported across plasma membranes^29^. The activity of cisplatin was mainly dominated by the stability of cisplatin. It had been reported that cisplatin was easily hydrolyzed^30^. The hydrolysis process released two chloride ions into water. The presence of chloride ions in water would increase the solution conductivity. Therefore, to evaluate the stability of cisplatin in DI and PIA water, the cisplatin solutions (0.28 mM) were prepared, and the conductivities were measured with time at 25 °C (Fig. 8). The conductivity of fresh cisplatin solution in PIA water (0.274 μS cm^−1^) was higher than that in DI water (0.184 μS cm^−1^). Mindfully, the higher conductivity of cisplatin solution in as-prepared PIA water was not attributed to the higher degree of cisplatin’s hydrolysis due to the intrinsically high conductivity of PIA water. With the increase of storage time, the conductivities of both solutions increased gradually, indicating that the cisplatin were hydrolyzed in both solutions. By plotting the relation of conductivity to time, two linear plots were obtained from DI water-based cisplatin and PIA water-based cisplatin solutions. The slope of PIA water-based cisplatin solution was 0.027 which was lower than that of DI water-based cisplatin solution (0.038). It indicated that the PIA water could avoid the hydrolysis of cisplatin, thus enhancing its stability. The high stability of cisplatin in PIA water could express the high activity of cisplatin in LLC-1 further. Therefore, higher cisplatin activity could be maintained when it was dissolved in PIA water.

**Table 1:** Analysis of the metastasis rate and survival time of LLC-1 xenograft mice.

	All cases (%)	Metastasis (%)^a^	*p* value^b,c^
Total	31 (100%)	5 (16.1%)	
Water type			0.048
DI	17 (54.8%)	5 (100%)	
PIA	14 (45.1%)	0 (0%)	
	**Survival days (mean ± SD)**		
Total	6.34 ± 0.41		
Treatment			
DI	4.62 ± 0.71		
DI + Cs	6.38 ± 0.61		0.081
PIA	6.57 ± 0.66		0.118
PIA + Cs	8.01 ± 0.77		0.009

^a^Lung metastasis was examined by gross observation of the whole lung.

^b^*p* values were analyzed by a two-tailed Fisher test.

^c^*p* values were analyzed by a log-rank test compared to the DI (DI water alone, n = 9) group and DI + Cs (DI water plus cisplatin, n = 8), PIA (PIA water, n = 7), or PIA + Cs (PIA water plus cisplatin, n = 7) group.

In this study, LLC-1 cells were used to clarify the biological effects of PIA water on NSCLC cells *in vitro* and *in vivo*. During *in vitro* incubation, PIA water-prepared culture medium had no observed antitumor effect on LLC-1 cells alone or with cisplatin treatment. Interestingly, PIA water-fed LLC-1-implanted B6 mice had less lung metastasis of LLC-1 tumors compared to mice fed DI water. This result suggests that PIA water may have a systemic biological effect that alters the tumor microenvironment, which was shifted against proliferation and/or metastasis of LLC-1 cells. Since the proinflammatory status of the tumor microenvironment contributes to tumor progression including metastasis of NSCLC, the anti-inflammatory property of PIA water may therefore delay tumor progression by suppressing the inflammation level in the tumor microenvironment of LLC-1-formed tumors. Furthermore, the overall survival time was also significantly prolonged in PIA water-fed mice with cisplatin administration. This suggests that PIA water can serve as integrated treatment to improve clinical outcomes of conventional chemotherapeutic agents, such as cisplatin, in NSCLC and other cancers. Although the *in vivo* study indicated that PIA water decreased the lung metastasis rate and improved the overall survival time of LLC-1-implanted mice, the present observations are still very limited. Further investigations to assess the antitumor efficiency and identify the biological mechanism mediated by PIA water as well as the potential adverse effects are therefore required.

In summation, we further clarified that PIA water mediated oxidative stress by inducing expression of an antioxidant factor, *Nrf2*. This PIA water-activated *Nrf2* expression may respond to the anti-inflammatory property of PIA water *in vitro*. In order to clarify the possible clinical application of PIA water to chronic inflammation-related diseases, an NSCLC mouse model was used for evaluating the therapeutic effects of PIA water in the preclinical stage. In NSCLC-grafted mice, PIA water not only decreased the lung metastasis rate, but also promoted the overall survival time with cisplatin administration. Taken together, these results suggest that PIA water with its anti-inflammatory property may serve as an alternative or integrative approach for clinical control of inflammation-related chronic diseases.

## Methods

### Materials

Electrolytes of NaCl and the reagents L-ascorbic acid, 5,5-dimethyl-1-pyrroline N-oxide (DMPO) were purchased from Sigma-Aldrich Organics (St. Louis, MO, USA). H_2_O_2_ and iron(II) chloride tetrahydrate were purchased from Acros Organics. Phosphate-buffered saline (PBS) was purchased from Bioman Organics. Ethylenediaminetetraacetic acid (EDTA) was purchased from Bioshop Organics. All of the reagents were used as received without further purification. All of the solutions were prepared using deionized (DI) 18.2-MΩ cm water provided from a Milli-Q system. All of the experiments were performed in an air-conditioned room at ca. 24 °C.

### Preparation of PIA water

PIA water was prepared using a previous method^15^. Typically, DI water (pH 6.95, T = 22.9 °C) was passed through a glass tube filled with AuNP-adsorbed ceramic particles under resonant illumination with green light-emitting diodes (LEDs, with wavelength maxima centered at 530 nm). Then the PIA water (pH 6.96, T = 23.5 °C) was collected in glass sample bottles for subsequent use within 2 h.

### Preparation of free hydroxyl radicals

Free hydroxyl radicals were obtained using the well-known Fenton reaction, in which ferrous iron donates an electron to hydrogen peroxide to produce the free hydroxyl radical. Because the produced free hydroxyl radicals were very unstable, they were capped by spin-trapping using DMPO to form more-stable complex radicals for exact detection. The sample preparation is described as follows. First, 140 μL DI water or PIA water was added to a microtube (Eppendorf). Then 20 μL PBS (10x) was added to the tube. A complex of EDTA-chelated iron(II) was prepared by mixing equal volumes of 0.5 mM iron(II) chloride tetrahydrate and 0.5 mM EDTA. Subsequently, 20 μL EDTA-chelated iron(II) (0.25 mM), 10 μL H_2_O_2_ (0.2 mM), and 10 μL DMPO (2 M) were sequentially added to the tube. The final volume in the tube was 200 μL. Exactly 1.5 min after the addition of DMPO, an electron spin resonance (ESR) analysis was performed. To obtain an ESR spectrum, a sample was scanned for ca. 1.5 min, accumulated eight times, and all signals were averaged.

### Measurement of free radicals by ESR spectroscopy

For ESR measurements, a Bruker EMX ESR spectrometer was employed. ESR spectra were recorded at room temperature using a quartz flat cell designed for solutions. The dead times between sample preparation and ESR analysis were exactly 1.5 and 10 min for experiments on hydroxyl and DPPH free radicals, respectively, after the last addition. Conditions of ESR spectrometry were as follows: 20 mW of power at 9.78 GHz, with a scan range of 100 G and a receiver gain of 6.32 × 10^4^.

### Determination of H_2_O_2_ in DI and PIA waters

A H_2_O_2_ standard curve was produced using an H_2_O_2_ assay kit (BioVision, Milpitas, CA, USA), and the corresponding optical density (OD) was measured at 570 nm. For this measurement, DI water, which was used to dilute the H_2_O_2_, was replaced with PIA water to evaluate its ability to scavenge H_2_O_2_. In experiments, 100 μL of H_2_O_2_ (1 mM) was diluted by adding 900 μL of DI water or PIA water before sampling 25 μL of above diluted solution into 96-well plate. Therefore, the volume ratio of H_2_O_2_ to PIA water is 1/9.

### Cell culture and treatment

HGFs were obtained from the American Type Culture Collection (Manassas, VA, USA). HGFs were maintained in Dulbecco’s modified Eagle’s medium (DMEM) (Gibco, Grand Island, NY, USA; cat. no. 11995-065 500 mL) supplied with 15% fetal bovine serum (FBS), 100 U/ml of penicillin, and 100 μg/ml of streptomycin. HGFs at 10^5^ per six wells were exposed to serum-free media prepared with DI or PIA water (containing 100 U/ml penicillin and 100 μg/ml streptomycin) for 0, 3, 6, and 9 h.

To assess the chemotherapeutic drug effect of PIA water on cancer cells *in vitro*, LLC-1 cells were seeded into a 96-well plate at 5 * 10^3^ cells per well for overnight incubation. Cells were then treated with 0–50 µM cisplatin for 48 h in culture medium prepared with DI or PIA water. Cell viability was determined by a 3-(4,5-dimethylthiazol-2-yl)-2,5-diphenyltetrazolium bromide (MTT) assay. The culture medium of LLC-1 was DMEM (Gibco) prepared with DI or PIA water, and supplied with 10% FBS (Gibco) and a mixture of 100 U/ml of penicillin and 100 μg/ml of streptomycin (Invitrogen Life Technologies, Carlsbad, CA, USA).

### Quantitative real-time polymerase chain reaction (qPCR)

To examine messenger (m)RNA expression, total RNA was extracted followed manufacturer’s instructions of the RNeasy Mini Kit (Qiagen). One microgram of total RNA was reverse-transcribed with a reverse transcription kit (Thermo-Fisher Scientific, Waltham, MA, USA) into complementary (c)DNA, and used as the template for real-time PCR reactions and analyses. The real-time PCRs were performed using SYBR Green reagent (Bio-Rad, Hercules, CA, USA) on CFX-Real-Time qPCR (Bio-Rad). The cDNA amount was analyzed by a qPCR with SYBR Green reagent (Bio-Rad) according to manufacturer’s instructions and used ΔΔCt to evaluate the relative multiples of change between the target gene and internal control, GAPDH. Primers used for the qPCR are indicated as follow:*Nrf2* (sense) 5′-CGCTTGGAGGCTCATCTCACA,*Nrf2* (antisense) 5′-CATTGAACTGCTCTTTGGACATCA; and *GAPDH* (sense) 5′-CGA CAG TCA GCC GCA TCT TCT TT -3′ and *GAPDH* (antisense) 5′-GGC AAC AAT ATC CAC TTT ACC AGA G -3′. This involved an initial denaturation at 95 °C for 5 min, followed by 40 cycles of denaturing at 95 °C for 5 s and combined annealing/extension at 60 °C for 10 s, as described in the manufacturer’s instructions.

### The transpleural orthotopic lung cancer model using LLC-1 cells

In total, 31 male, 6-week-old B6 mice were purchased from the National Laboratory Animal Center (NLAC, Taipei, Taiwan), and housed for 1 week for environment adaptation under specific pathogen-free conditions in the Laboratory Animal Center, Taipei Medical University. All animal experimental protocols were approved by the Institutional Animal Care and Use Committee (LAC-2014-0106) of Taipei Medical University. We confirmed that the animal experiment described in this manuscript was approved by an appropriate institute (IACUC approval no: LAC-2014-0106, as shown in manuscript), and also performed in accordance with the relevant guidelines and regulations. Mice were further divided into two groups with DI water (*n* = 17) or PIA water (*n* = 14) supplied *ad libitum* for a 1-week duration. Before LLC-1 cell implantation, each mouse received 5 * 10^5^ LLC-1 cells which were suspended in a 50-µL mixture of culture medium and BD Matrigel^TM^ basement membrane matrix (BD Biosciences, San Jose, CA, USA) in a 1:1 ratio by an intercostal injection along the median axillary line in the left lung. After the LLC-1 cell injection, mice were housed for a 1-week duration for tumor development, and then administered a single intraperitoneal (i.p.) injection of 5 mg/kg cisplatin until the tenth day^31^. Mice that survived to the tenth day were sacrificed by CO_2_ euthanasia. The whole lung of each mouse was grossly observed to examine metastasis of tumor lesions on the lung lobes, and these were further identified by hematoxylin and eosin (HE) staining. The animal experimental plan is shown in Fig. S2.

### Statistical analysis

Analyses of metastasis and overall survival were performed with SPSS software (SPSS, Chicago, IL, USA). The metastasis incidence between mice that received DI or PIA water was compared by a two-tailed Fisher’s exact test. Overall survival was estimated using a Kaplan-Meier survival analysis, and the survival time between groups was compared using the log-rank test.

## Electronic supplementary material

Supplementary Information
