# Supplementary material for: Innovatively Therapeutic Strategy on Lung Cancer by Daily Drinking Antioxidative Plasmon-Induced Activated Water
Source: Sci Rep. 2018 Apr 20;8:6316. doi: 10.1038/s41598-018-24752-x (PMC5910416; doi:10.1038/s41598-018-24752-x)
Supplement: Supplementary file 1 — Supplementary Information [file 41598_2018_24752_MOESM1_ESM.doc]

Innovatively Therapeutic Strategy on Lung Cancer by Daily Drinking Antioxidative Plasmon-Induced Activated Water

Chien-Kai Wang^1,2^, Hsiao-Chien Chen^3^, Sheng-Uei Fang ^4,5^, Chia-Wen Ho^6^, Cheng-Jeng Tai^2,5^, Chih-Ping Yang^3^ & Yu-Chuan Liu^3^,*

^1^ Department of Animal Science, National Chung Hsing University, No. 250, Guoguang Rd., Taichung 402, Taiwan. ^2^ Division of Hematology and Oncology, Department of Internal Medicine, Taipei Medical University Hospital, No. 252, Wuxing St., Taipei 11031, Taiwan. ^3^ Department of Biochemistry and Molecular Cell Biology, School of Medicine, College of Medicine, Taipei Medical University, No. 250, Wuxing St., Taipei 11031, Taiwan. ^4^ Division of Gastroenterology and Hepatology, Department of Internal Medicine, Taipei Medical University Hospital, No. 252, Wuxing St., Taipei 11031, Taiwan. ^5^ Department of Internal Medicine, School of Medicine, College of Medicine, Taipei Medical University, No. 250, Wuxing St., Taipei 11031, Taiwan. ^6^ Center for Cancer Research, Taipei Medical University, No. 250, Wuxing St., Taipei 11031, Taiwan.

C.-K. Wang, H.-C. Chen. and S.-U. Fang contributed equally to this work. Correspondence and requests for materials should be addressed to Y.-C. Liu, (E-mail: [liuyc@tmu.edu.tw](mailto:liuyc@tmu.edu.tw)).

Supplementary Information


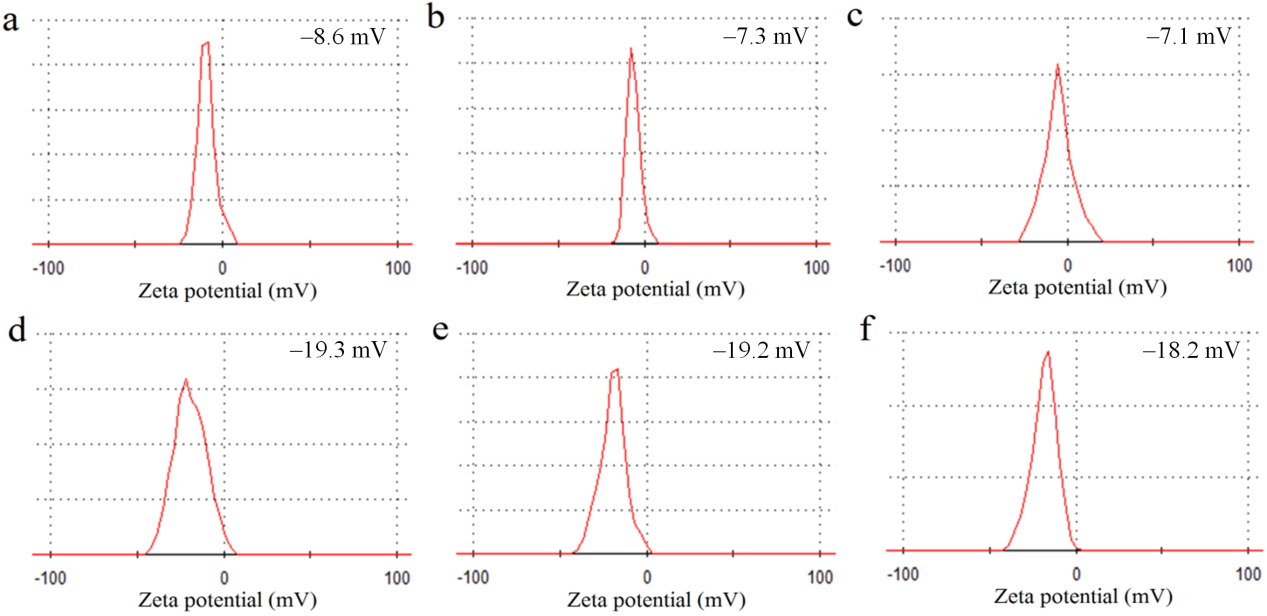


**Figure S1.** The charge of cisplatin solution. The time-dependent zeta potentials of cisplatin dissolved in (a~c) DI and (d~f) PIA waters with 0.5 % NaCl.


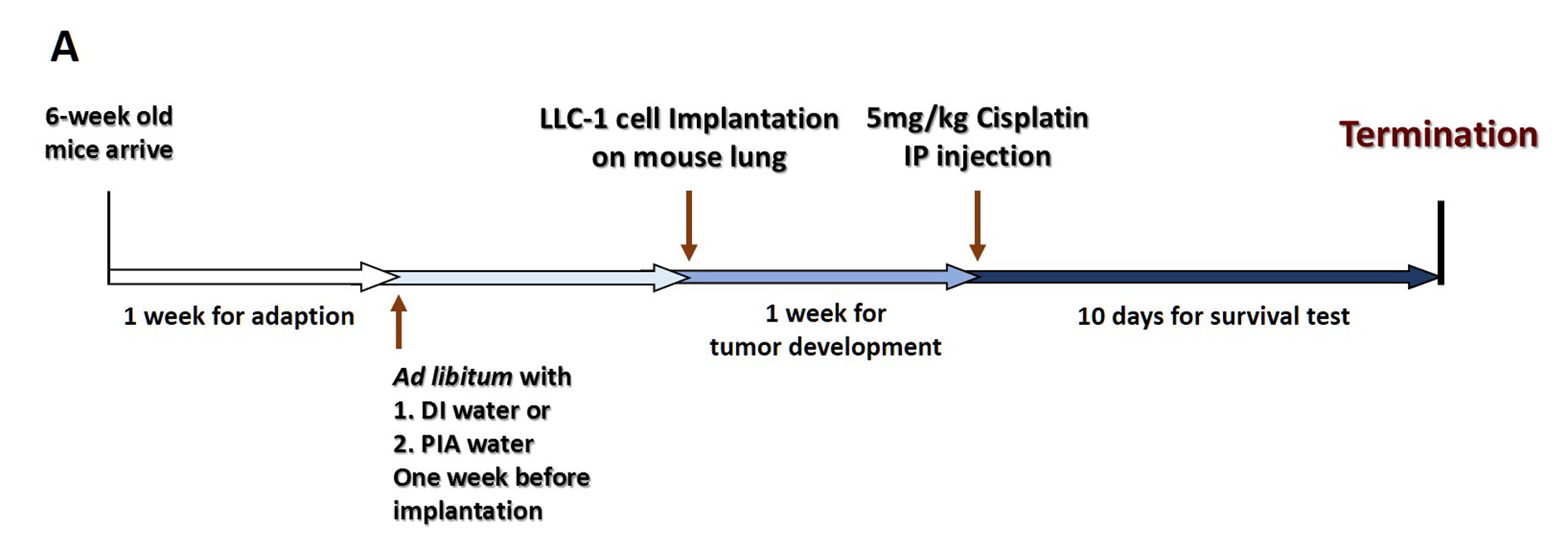


**Figure S2.** The animal experiment plan of transpleural orthotopic lung cancer model using LLC-1 cells.
